# COVID-19: Putting the General Data Protection Regulation to the Test

**DOI:** 10.2196/19279

**Published:** 2020-05-29

**Authors:** Stuart McLennan, Leo Anthony Celi, Alena Buyx

**Affiliations:** 1 Institute of History and Ethics in Medicine Technical University of Munich Munich Germany; 2 Division of Pulmonary, Critical Care, and Sleep Medicine Beth Israel Deaconess Medical Center Boston, MA United States; 3 Harvard–Massachusetts Division of Health Science and Technology Cambridge, MA United States

**Keywords:** COVID-19, data sharing, GDPR, research exemption, global health, public health, research, digital health, electronic health records, EHR

## Abstract

The coronavirus disease (COVID-19) pandemic is very much a global health issue and requires collaborative, international health research efforts to address it. A valuable source of information for researchers is the large amount of digital health data that are continuously collected by electronic health record systems at health care organizations. The European Union’s General Data Protection Regulation (GDPR) will be the key legal framework with regard to using and sharing European digital health data for research purposes. However, concerns persist that the GDPR has made many organizations very risk-averse in terms of data sharing, even if the regulation permits such sharing. Health care organizations focusing on individual risk minimization threaten to undermine COVID-19 research efforts. In our opinion, there is an ethical obligation to use the research exemption clause of the GDPR during the COVID-19 pandemic to support global collaborative health research efforts. Solidarity is a European value, and here is a chance to exemplify it by using the GDPR regulatory framework in a way that does not hinder but actually fosters solidarity during the COVID-19 pandemic.

As the severe acute respiratory syndrome coronavirus 2 (SARS-CoV-2) continues to spread around the globe, researchers are racing to understand and contain the pandemic, learn how to best treat patients with SARS-CoV-2 infection and the resulting coronavirus disease (COVID-19), and develop a vaccine. The COVID-19 pandemic is also very much a global health issue and requires collaborative, international health research efforts to address it. A valuable source of information for researchers is the large amount of digital health data that are continuously collected by the electronic health record systems of health care organizations. However, such digital health data typically exists in separate systems and researchers in many countries are currently severely hamstrung by the lack of integrated and comprehensive, publicly available, patient-level data regarding COVID-19. They are having to derive answers from limited analyses of small case series, while large amounts of relevant digital health data sits unexamined on hospital servers around the world. This situation has led to calls for a common, multinational, COVID-19 database to be created, pointing to the Medical Information Mart for Intensive Care (MIMIC) database at the Beth Israel Deaconess Medical Center in Boston as a model for publicly sharing deidentified electronic health data [1].

While setting up COVID-19-related databases for research makes obvious sense from a research perspective, there is also currently a broader societal reason why this is a good idea. Indeed, the COVID-19 pandemic has put solidarity into strong focus; many ongoing measures to contain the spread have been described as solidarity practices—that is, as prosocial behaviors to help and/or protect others, or collective resources such as health care systems, that are based on the recognition of a shared interest. Health databases and biobanks have also previously been framed as solidarity-based endeavors, and solidarity-based governance models have been proposed to reflect the prosocial motivation many people have toward such resources, which at the same time avoid some of the burden of the usual restrictive, autonomy-based governance models [2].

As the total deaths from COVID-19 continues to increase globally, the ethical and social imperative to quickly curtail the pandemic is clear. However, this does not negate the need for the use of digital health data to respect data protection regulations and patient privacy and confidentiality [3]. In fact, although the scale of COVID-19 is clearly new, the ethical challenge of balancing confidentiality with public health has been well discussed [4-7].

With the epicenter of the pandemic currently shifting from Europe to the United States, the European Union’s (EU) General Data Protection Regulation (GDPR) will be the key legal framework with regard to using and sharing European digital health data for research purposes [8]. However, concerns persist that the GDPR has made many organizations very risk-averse in terms of data sharing, even if the regulation permits such sharing. Health care organizations focusing on individual risk minimization threaten to undermine COVID-19 research efforts.

The European Data Protection Board has stressed the importance of protecting personal data during the COVID-19 pandemic. However, it has also noted: “Data protection rules (such as GDPR) do not hinder measures taken in the fight against the coronavirus pandemic” [9]. Indeed, article 9(2)(i) of the GDPR explicitly allows the processing of sensitive personal data (including genetic data, biometric data, and data concerning health) if it is “necessary for reasons of public interest in the area of public health.” Recitals 46, 52, 53, and 54 also explicitly acknowledge the need to sometimes process special categories of personal data for reasons of public interest in the area of public health.

Furthermore, article 9(2)(j) sets out a scientific research exemption for the processing of sensitive personal data, which could occur without consent if subject to appropriate safeguards, which may include pseudonymization (deidentification) (see article 89(1)) (Table 1). Researchers and health care organizations wanting to utilize and share patient-level data regarding COVID-19 from data subjects residing in the EU will need to be aware of the following:

- The GDPR applies to any personal data concerning an identified or identifiable natural person, but not to anonymous information. As the GDPR does not distinguish between anonymized and anonymous data, databases collecting identifiable data for research purposes will be excluded from the scope of the GDPR if the data are later rendered anonymized [8,10].
- Pseudonymized data is now recognized as personal data if it could be attributed to a natural person by the use of additional information. Given pseudonymized health data is what health care databases typically use, recognizing pseudonymized data as personal data may result in more bureaucracy, particularly for those countries that currently consider pseudonymized data to fall outside the scope of personal data [8,10].
- The processing of special categories of personal data (“sensitive personal data”), including genetic data, biometric data, and data concerning health, shall be prohibited under the GDPR unless certain conditions applies. Health care databases using pseudonymized sensitive personal data will need to either obtain explicit consent from the data subject or for the data to be processed under the scientific research exemption set out in the GDPR, which could occur without consent if subject to appropriate technical and organizational safeguards [8,10].

In our opinion, there is an ethical obligation to use the GDPR scientific research exemption clause during the COVID-19 pandemic to support global collaborative health research efforts. However, while the provision is there, researchers and research institutions in Europe have been reluctant to use it, likely due to fear of the difficulties that may be caused by their national bodies. In fact, consortia funded in the current H2020 funding scheme by the European Commission have overwhelmingly used other more burdensome legal justifications, such as informed consent, than the research exemption.

This is not sufficient for the current situation. COVID-19 is a real test for the GDPR. There is a strong ethical case that countries use the regulatory leeway the GDPR provides for enabling health data to be used for research purposes and that they support health care organizations and investigators to invoke the research exemption confidently in the context of a global pandemic. Recent research in some European countries also suggests that many people would accept the secondary use of their data for health-related research under the research exemption, based on prosocial motivations such as solidarity [11]. Solidarity is a European value, and here is a chance to exemplify it by using the GDPR regulatory framework in a way that does not hinder but actually fosters solidarity during the COVID-19 pandemic.

**Table 1:** Scientific research exemption provisions of the European Union’s General Data Protection Regulation (GDPR).

GDPR article	Relevant sections
Article 9: Processing of special categories of personal data	Section 1: Processing of personal data revealing racial or ethnic origin, political opinions, religious or philosophical beliefs, or trade union membership, and the processing of genetic data, biometric data for the purpose of uniquely identifying a natural person, data concerning health or data concerning a natural person’s sex life or sexual orientation shall be prohibited. Section 2: Paragraph 1 shall not apply if one of the following applies: The data subject has given explicit consent to the processing of those personal data for one or more specified purposes, except where Union or Member State law states that the prohibition referred to in paragraph 1 may not be lifted by the data subject; …. Processing is necessary for reasons of public interest in the area of public health, such as protecting against serious cross-border threats to health or ensuring high standards of quality and safety of health care and of medicinal products or medical devices, on the basis of Union or Member State law which provides for suitable and specific measures to safeguard the rights and freedoms of the data subject, in particular professional secrecy; (j) Processing is necessary for archiving purposes in the public interest, scientific or historical research purposes or statistical purposes in accordance with Article 89(1) based on Union or Member State law which shall be proportionate to the aim pursued, respect the essence of the right to data protection and provide for suitable and specific measures to safeguard the fundamental rights and the interests of the data subject.
Article 89: Safeguards and derogations relating to processing for archiving purposes in the public interest, scientific or historical research purposes, or statistical purposes	Section 1: Processing for archiving purposes in the public interest, scientific or historical research purposes, or statistical purposes shall be subject to appropriate safeguards, in accordance with this Regulation, for the rights and freedoms of the data subject. Those safeguards shall ensure that technical and organisational measures are in place in particular in order to ensure respect for the principle of data minimisation. Those measures may include pseudonymisation provided that those purposes can be fulfilled in that manner. Where those purposes can be fulfilled by further processing which does not permit or no longer permits the identification of data subjects, those purposes shall be fulfilled in that manner.

## Abbreviations

COVID-19: coronavirus disease
EU: European Union
GDPR: coronavirus disease
SARS-CoV-2: severe acute respiratory syndrome coronavirus 2
